# Neuroprotective Effects of β-Caryophyllene against Dopaminergic Neuron Injury in a Murine Model of Parkinson’s Disease Induced by MPTP

**DOI:** 10.3390/ph10030060

**Published:** 2017-07-06

**Authors:** Juan M. Viveros-Paredes, Rocio E. González-Castañeda, Juerg Gertsch, Veronica Chaparro-Huerta, Rocio I. López-Roa, Eduardo Vázquez-Valls, Carlos Beas-Zarate, Antoni Camins-Espuny, Mario E. Flores-Soto

**Affiliations:** 1 Departamento de Farmacobiología CUCEI, Universidad de Guadalajara, 44430 Guadalajara, Mexico; jviveros99@hotmail.com (J.M.V.-P.); rlopezroa@gmail.com (R.I.L.-R.); 2 Laboratorio de Microscopía de Alta Resolución, Departamento de Neurociencias, Centro Universitario de Ciencias de la Salud, Universidad de Guadalajara, 44340 Guadalajara, Mexico; roglezca@yahoo.com.mx; 3 Institute of Biochemistry and Molecular Medicine, NCCR Trans Cure, University of Bern, CH-3012 Bern, Switzerland; gertsch@ibmm.unibe.ch; 4 Laboratorio de Neurobiología Celular y Molecular, Centro de Investigación Biomédica de Occidente (CIBO), Instituto Mexicano del Seguro Social, 44421 Guadalajara, Mexico; veronicach73@mail.com; 5 Laboratorio de Inmunodeficiencias y Retrovirus Humanos, Centro de Investigación Biomédica de Occidente, Instituto Mexicano del Seguro Social, 44421 Guadalajara, Mexico; evazquez@cencar.udg.mx; 6 Laboratorio de Regeneración y Desarrollo Neural, Instituto de Neurobiología, Departamento de Biología Celular y Molecular, CUCBA, Universidad de Guadalajara, 44340 Guadalajara, Mexico; carlosbeas55@gmail.com; 7 Unitat de Farmacologia i Farmacognòsia, Facultat de Farmàcia i Ciencias de l’Alimentació, Universitat de Barcelona, 08028 Barcelona, Spain; camins@ub.edu; 8 Biomedical Research Networking Center in Neurodegenerative Diseases (CIBERNED), 28031 Madrid, Spain

**Keywords:** β-caryophyllene, Parkinson’s disease, MPTP, microglial activation

## Abstract

Parkinson’s disease (PD) is one of the most common neurodegenerative disorders and is characterized by the loss of dopaminergic neurons in the substantia nigra (SN). Although the causes of PD are not understood, evidence suggests that its pathogenesis is associated with oxidative stress and inflammation. Recent studies have suggested a protective role of the cannabinoid signalling system in PD. β-caryophyllene (BCP) is a natural bicyclic sesquiterpene that is an agonist of the cannabinoid type 2 receptor (CB2R). Previous studies have suggested that BCP exerts prophylactic and/or curative effects against inflammatory bowel disease through its antioxidative and/or anti-inflammatory action. The present study describes the neuroprotective effects of BCP in a 1-methyl-4-phenyl-1,2,3,6-tetrahydropyridine (MPTP)-induced murine model of PD, and we report the results of our investigation of its neuroprotective mechanism in neurons and glial cells. In the murine model, BCP pretreatment ameliorated motor dysfunction, protected against dopaminergic neuronal losses in the SN and striatum, and alleviated MPTP-induced glia activation. Additionally, BCP inhibited the levels of inflammatory cytokines in the nigrostriatal system. The observed neuroprotection and inhibited glia activation were reversed upon treatment with the CB2R selective antagonist AM630, confirming the involvement of the CB2R. These results indicate that BCP acts via multiple neuroprotective mechanisms in our murine model and suggest that BCP may be viewed as a potential treatment and/or preventative agent for PD.

## 1. Introduction

Parkinson’s disease (PD) is a common neurodegenerative disorder characterized by the progressive degeneration of nigrostriatal dopaminergic (DA) neurons. The most prominent biochemical changes in PD involve the reduction of striatal dopamine levels, which may result in abnormal motor behaviour, including resting tremors, rigidity, and bradykinesia [1]. The incidence of PD increases with age [2], and PD affects approximately 6% of those over the age of 65. Even in the beginning stages, PD can lead to significant dysphagia that negatively impacts the quality of life [3]. The primary neurodegeneration trigger remains uncertain; however, several pathophysiological mechanisms have been implicated, including ageing, highly dense microglia in the substantia nigra (SN), inflammation, oxidative/nitrosative stress, abnormal protein deposits, and decreased neurotrophic factors [4]. These mechanisms lead to microglia activation, which, in turn, favours an oxidative and inflammatory environment that is facilitative of neuronal death [5]. In the substantia nigra of PD patients and in 1-methyl-4-phenyl-1,2,3,6-tetrahydropyridine (MPTP) models of PD, key enzymes involved in reactive oxygen species (ROS) production, such as microglia NAPDH oxidase, are upregulated in damaged areas and contribute to DA neuronal cell death [6]. In addition, proinflammatory cytokines, such as IL-1βand TNF-α, are augmented in PD patients [7,8] and participate in DA neuronal cell death in the MPTP model of PD [9,10].

Recent studies have suggested that the cannabinoid signalling system plays a protective role in PD. For example, during PD, endocannabinoids accumulate, cannabinoid receptors are upregulated, and treatment with cannabinoid agonists, either endocannabinoids or phytocannabinoids/synthetic cannabinoids, protects nigrostriatal dopaminergic neurons against MPTP neurotoxicity by inhibiting microglia activation [11].

The effects of cannabinoids are mainly mediated by the action of G-protein-coupled type 1 cannabinoid receptors (CB1Rs) and type 2 cannabinoid receptors (CB2Rs). CB2Rs were initially thought to be expressed primarily in activated microglia and peripheral immune cells where they regulate cytokine/chemokine production and cell migration [12]. Numerous in vitro studies have shown that microglia activation of the CB2R inhibits the release of proinflammatory cytokines [13] and increases the release of anti-inflammatory cytokines. On the other hand, in vivo studies have shown that pharmacological activation of the CB2R can reduce the activation of microglia cells and inhibit dopaminergic cell death in the substantia nigra in PD models [14]. Moreover, genetic studies have supported these pharmacological findings, with CB2R knock-out mice displaying increased microglia activation, neural pathology and functional deficits in models of PD [15]. Post-mortem studies of human patients with PD have revealed that the CB2R levels were elevated in microglial cells in the substantia nigra of PD patients [16]. Although a great amount of evidence has indicated the presence of functional CB2Rs in the central nervous system (CNS), potential neuroprotective properties of these CB2Rs and the molecular mechanisms underlying the effects associated with their activation during PD are not yet understood. In addition, an important advantage of compounds that specifically activate CB2R is that they do not cause any of the effects frequently associated with psychotropic cannabinoids that activate the CB1R, indicating that can be safe and well-tolerated in clinical applications.

β-Caryophyllene (BCP) is a natural bicyclic sesquiterpene isolated from clove leaf oil, clove stem oil, cinnamon leaf oil, and pine oil fractions. It has been used as a flavouring agent and as a fragrance ingredient since the 1930s [17]. Further, it has been granted the generally recognized-as-safe (GRAS) status by the Flavour and Extract Manufacturers Association and is approved by the U.S. FDA for use in food because of its low toxicity. This compound is known to be antimicrobial [18], antioxidant [19] and anticarcinogenic [20], and it also possesses properties that enhance skin penetration [21]. Specifically, essential oils that have BCP as a major component (30.6%) have shown obvious anti-inflammatory activity against carrageenan- and prostaglandin E (PGE) 1-induced oedema in rats as well as antiarthritic activity [22,23]. Recently, it has been demonstrated that BCP exerts prophylactic and/or curative effects against inflammatory bowel disease through its antioxidative and/or anti-inflammatory action [24]. Like other CB2R agonists, *trans*-caryophyllene inhibits inflammation, oedema formation, and the expression of inflammatory mediators in vitro and in vivo. Thus, we hypothesized that BCP may improve motor behaviour and prevent the loss of nigrostriatal dopaminergic neurons by inhibiting brain inflammation and oxidative stress in an experimental model of Parkinson’s disease. However, the influence of BCP on PD has not yet been explored.

## 2. Results

### 2.1. Motor Behavioural Test: β-Caryophyllene Inhibits Chronic MPTP-Induced Defects in Motor Coordination via the CB2 Receptor

#### 2.1.1. Pole Test Analysis

**Figure 1:**
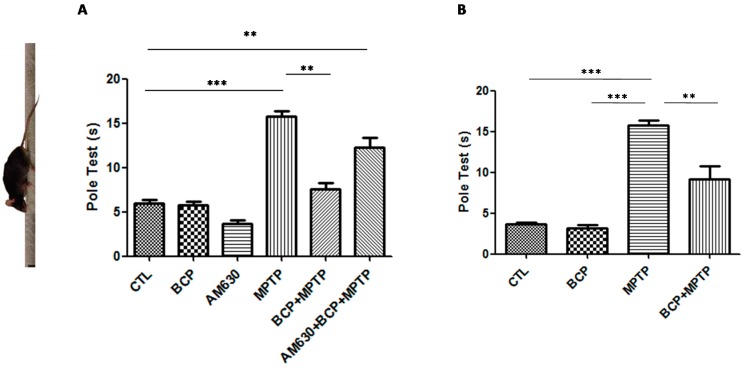
The pole test. The protective effects of BCP administered both intraperitoneally (**A**) and orally (**B**) against MPTP-induced behavioural impairment in mice. MPTP-treated mice displayed a significant increase in the total time required to traverse the pole 3 d post lesion relative to that of the CTL-injected mice. The data are presented as mean ± SEM of six individual experiments. Intraperitoneal: *** *p* < 0.001, CTL vs. MPTP. ** *p* < 0.01, MPTP vs. BCP + MPTP. ** *p* < 0.01, CTL vs. AM630 + BCP + MPTP treatment. Oral: *** *p* < 0.001, CTL vs. MPTP. ** *p* < 0.01, MPTP vs. BCP + MPTP. *** *p* < 0.001, BCP vs. MPTP treatment.

The effects of BCP administered both intraperitoneally and orally on mouse motor function were assessed with the pole test. Mice were examined under baseline conditions prior to the administration of MPTP. Deficits in motor coordination were clearly observed in the MPTP-treated mice, with the most severe motor function impairment being 16.5 ± 1.7 s required for the mice to turn at the top of the pole and climb down in comparison to that of the control group. Administration of 10 mg/kg BCP both orally and intraperitoneally prior to the administration of MPTP significantly showed a recovery effect on the MPTP-induced movement impairment (intraperitoneal: 7.3 ± 1.5 s; oral: 5.7 ± 1.8 s). AM630 (3 mg/kg, i.p.) significantly reversed the BCP-induced improvement in the pole test (*p* < 0.01). In the groups treated only with BCP and AM630, no statistically significant changes compared to the control group were observed (Figure 1).

#### 2.1.2. Gait Test Analysis

**Figure 2:**
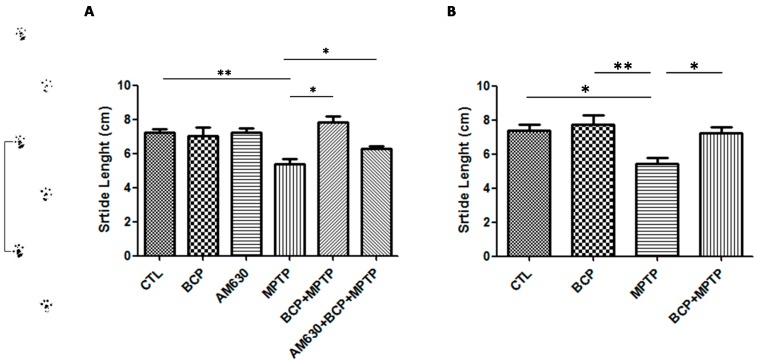
The gait test. The protective effects of BCP administered both intraperitoneally (**A**) and orally (**B**) against MPTP-induced behavioural impairment in mice. The MPTP-lesioned mice exhibited a significant decrease in the average stride length 3 days post-lesion compared to that of the CTL-treated mice. The data are presented as the mean ± SEM of six individual experiments. Intraperitoneal: ** *p* < 0.01, CTL vs. MPTP. * *p* < 0.05, MPTP vs. BCP + MPTP. * *p* < 0.05, MPTP vs. AM630 + BCP + MPTP treatment. Oral: * *p* < 0.05, CTL vs. MPTP. ** *p* < 0.01, BCP vs. MPTP. * *p* < 0.05, MPTP vs. BCP + MPTP treatment.

On the other hand, a shortened shift stride is considered a symptom of Parkinson’s disease. In the group treated with MPTP, decreased stride length relative to that of the control group was observed. However, in the groups treated with BCP (10 mg/kg, i.p.) administered both orally and intraperitoneally prior to MPTP administration, an increase in stride length compared to that of the MPTP-treated group was observed. AM630 (3 mg/kg, i.p.) significantly reversed the BCP-induced improvement in the gait test (*p* < 0.01). In the groups treated with only BCP or AM630, no statistically significant changes were observed compared to the control group (Figure 2).

#### 2.1.3. Beam Test

**Figure 3:**
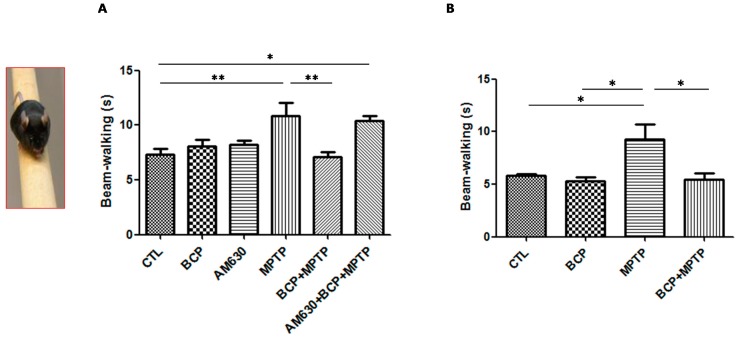
The beam test. The protective effects of BCP administered both intraperitoneally (**A**) and orally (**B**) against MPTP-induced behavioural impairment in mice. MPTP-lesioned mice exhibited a significant decrease in the average time required to cross the beam 3 days post-lesion compared to that of the CTL-treated mice. The data are presented as the mean ± SEM of six individual experiments. Intraperitoneal: ** *p* < 0.01, CTL vs. MPTP. ** *p* < 0.01, MPTP vs. BCP + MPTP. * *p* < 0.05, CTL vs. AM630 + BCP + MPTP treatment. Oral: * *p* < 0.05, CTL vs. MPTP. * *p* < 0.05, BCP vs. MPTP. * *p* < 0.05, MPTP vs. BCP + MPTP treatment.

Motor defects of animals from each of the treatment groups to navigate the balance beam was evaluated to study this aspect. Figure 3 shows that the average time it took the control mice to cross the beam was 8 s, while mice exposed to MPTP took 11.8 s to cross the beam, which was statistically significant compared to that of the group control. 

In contrast, in the groups treated with BCP (10 mg/kg, i.p.) administered both orally and intraperitoneally prior to the administration of MPTP, a decrease in the time it took the animals to cross the beam of 7.5 s intraperitoneally and 7.5 s orally was observed, which was statistically significant with respect to that of the MPTP treatment group. AM630 (3 mg/kg, i.p.) significantly reversed the BCP-induced improvement in the beam test (*p* < 0.01). In the groups treated with only BCP or AM630, no statistically significant changes were observed compared to the control group.

### 2.2. β-Caryophyllene Prevents Chronic MPTP-Induced Dopaminergic Neuron Loss in the SNpc and STR via the CB2 Receptor

**Figure 4:**
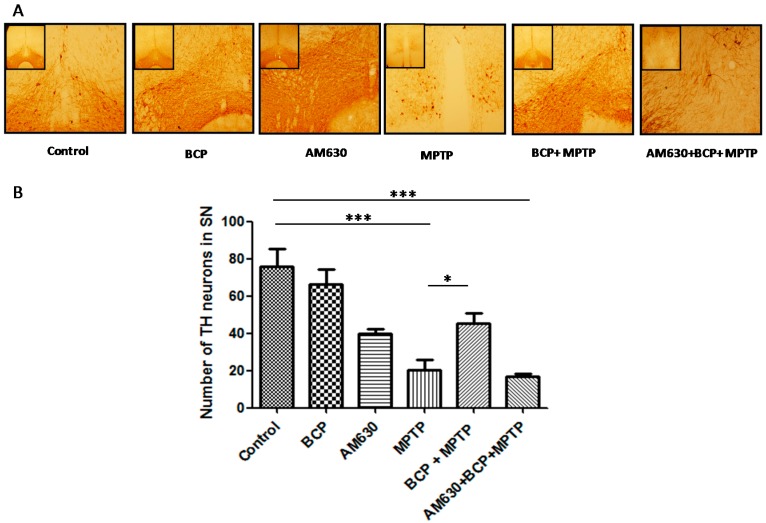
BCP treatment attenuates the MPTP-induced nigrostriatal dopaminergic neuronal damage. C57BL/6 mice were treated with MPTP for 5 days (30 mg/kg, i.p.). The mice were sacrificed on the 3rd day after MPTP injection after being subjected to behavioural tests. Photomicrographs of representative SN (**A**) sections stained with an antibody against TH. Reduced activity of TH-neurons was observed in the MPTP-treated mice, which was partially prevented by treatment with BCP. The number of TH-positive neurons in the SN (**B**) was expressed as the mean ± SEM of six individual experiments. *** *p* < 0.001, CTL vs. MPTP treatment. * *p* < 0.05, MPTP treatment vs. BCP + MPTP treatment. *** *p* < 0.001, CTL vs. AM630 + BCP + MPTP treatment.

TH-immunoreactivity was measured in the SNpc and STR to explore the effects of BCP on the MPTP-induced degeneration of dopaminergic neurons. Treatment with MPTP significantly reduced the number of TH-IR neurons by 74% compared with that of the control mice. However, in mice treated with BCP (10 mg/kg, i.p.) and MPTP, the number of dopaminergic neurons was reduced by only 18.4 ± 3.4% compared with that of the control. To determine whether the protective effect of BCP against MPTP-induced dopaminergic neuronal degeneration was mediated by CB2R, mice were pretreated with the CB2R selective antagonist AM630 (3 mg/kg, i.p.) 30 min prior to BCP administration. AM630 significantly reversed the protective effect of BCP, as evidenced by a reduction in the number of dopaminergic neurons (79 ± 1.7%) compared with that of the control mice (*p* < 0.01; Figure 4). Systemic administration of BCP or AM630 by themselves did not significantly affect the number of TH-immunoreactive cells (*p* < 0.05 compared with that of the controls).

**Figure 5:**
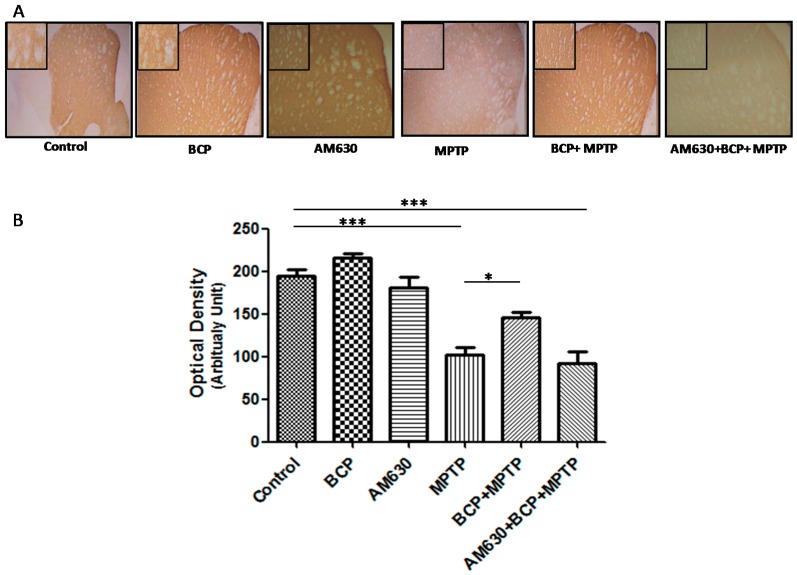
BCP treatment attenuates MPTP-induced nigrostriatal dopaminergic neuronal damage. C57BL/6 mice were treated with MPTP for 5 days (30 mg/kg, i.p.). The mice were sacrificed on the 3rd day after MPTP injection after being subjected to behavioural tests. Photomicrographs of representative STR (**A**) sections stained with an antibody against TH. Reduced activity of TH-neurons was observed in the MPTP-treated mice, which was partially prevented by treatment with BCP. The number of TH-positive fibres in the STR (**B**) was expressed as the mean ± SEM of six individual experiments. *** *p* < 0.001, CTL vs. MPTP treatment. * *p* < 0.05, MPTP treatment vs. BCP + MPTP treatment. *** *p* < 0.001, CTL vs. AM630 + BCP + MPTP treatment.

Densitometric analysis showed that the number of TH-positive fibres in the STR was decreased by 61% after MPTP administration compared to that of the control group (*p* < 0.001). By contrast, mice treated with BCP (10 mg/kg, i.p.) and MPTP displayed an increased number of TH-positive fibres in the STR compared to that of the MPTP group (*p* < 0.001). In mice treated with BCP and MPTP, the number of TH-positive fibres was reduced by only 17.4 ± 5.4% compared with that of the control mice. AM630 significantly reversed the protective effect of BCP, as evidenced by a reduction in the number of TH-positive fibres in the STR (47.7 ± 1.7%) compared with that of the control mice (*p* < 0.05; Figure 5). Systemic administration of BCP did not significantly affect the number of TH-positive fibres.

### 2.3. β-Caryophyllene Inhibits Chronic MPTP-Induced Astrocyte and Microglia Activation in the SNpc and STR via the CB2 Receptor

**Figure 6:**
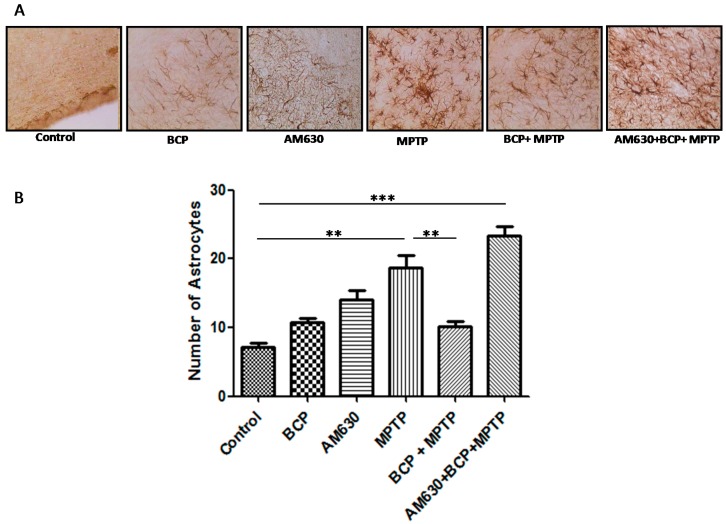
Effect of BCP on MPTP-induced astrocyte activation in the mouse SNpc. (**A**) Glial fibrillary acidic protein (GFAP)-IR astrocytes in mouse SNpc. Scale bar: 200 μm. (**B**) Stereological cell counts of GFAP-IR neurons. The data are presented as the mean ± SEM of six individual experiments. ** *p* < 0.01, CTL vs. MPTP treatment. ** *p* < 0.01, MPTP treatment vs. BCP + MPTP treatment. *** *p* < 0.001, CTL vs. AM630 + BCP + MPTP treatment.

**Figure 7:**
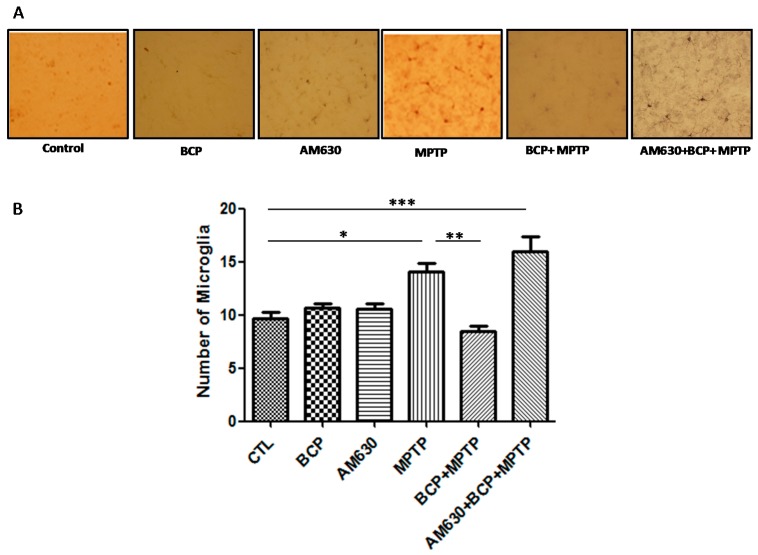
Effect of BCP on MPTP-induced microglia activation in the mouse SNpc. (**A**) Ionized calcium-binding adapter molecule-1 (IBA-1)-IR microglia in mouse SNpc. Scale bar: 200 μm. (**B**) Stereological cell counts of IBA-1-IR microglia. The data are presented as the mean ± SEM of six individual experiments. * *p* < 0.05, CTL vs. MPTP treatment. ** *p* < 0.01, MPTP vs. BCP + MPTP treatment. *** *p* < 0.001, CTL vs. AM630 + BCP + MPTP treatment.

To evaluate the effects of BCP on astrocyte and microglia activation, the astrocyte marker GFAP and the microglia marker IBA-1 were detected by immunohistochemistry. Chronic administration of MPTP (30 mg/kg, i.p.) markedly activated astrocytes (Figure 6) and microglia (Figure 7) in the mouse SNpc, as evidenced by significantly increased numbers of GFAP-IR cells (163.2% compared with that of the control mice) and IBA-1-IR cells (46.2% compared with that of the control mice).

Treatment with 10 mg/kg BCP (10 mg/kg, i.p.) suppressed the MPTP-induced increase in GFAP-IR cells and IBA-1-IR cells by 84.5 and 66.2%, respectively, (*p* < 0.01) compared with the effects of MPTP treatment alone. The protective effect of BCP against astrocyte activation and microglia was fully reversed by pre-treating with 3 mg/kg AM630. Systemic administration of BCP alone not significantly affect the numbers of GFAP-IR or IBA-1-IR cells (*p* < 0.05 compared with that of the control mice) (Figure 6 and Figure 7).

**Figure 8:**
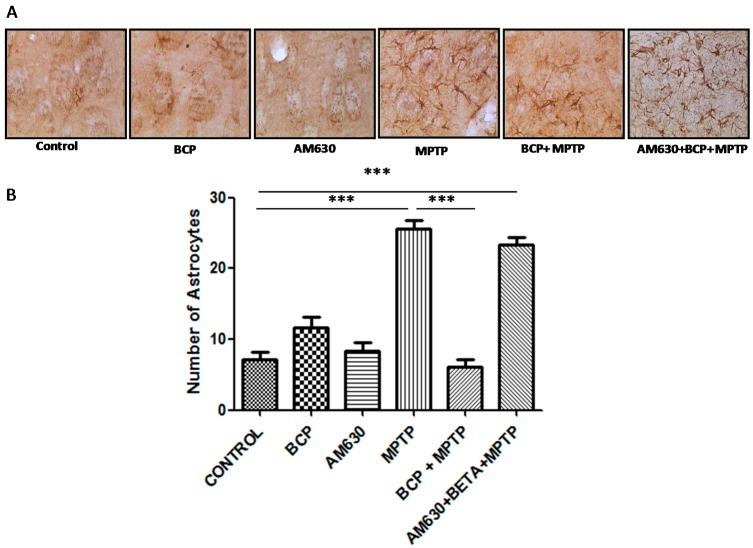
Effect of BCP on MPTP-induced astrocyte activation in the mouse STR. (**A**) Glial fibrillary acidic protein (GFAP)-IR astrocytes in mouse STR. Scale bar: 200 μm. (**B**) Stereological cell counts of GFAP-IR astrocyte. The data are presented as mean ± SEM of six individual experiments. *** *p* < 0.001, CTL vs. MPTP. *** *p* < 0.001, MPTP vs. BCP+ MPTP. *** *p* < 0.001, CTL vs. AM630 + BCP + MPTP treatment.

**Figure 9:**
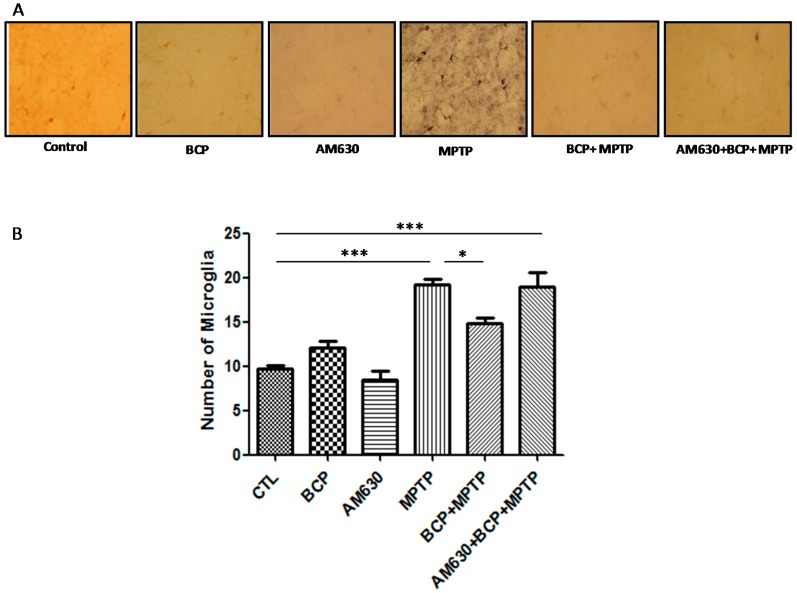
Effect of BCP on MPTP-induced microglia activation in the mouse STR. (**A**) Ionized calcium-binding adapter molecule-1 (IBA-1)-IR microglia in mouse STR. Scale bar: 200 μm. (**B**) Stereological cell counts of IBA-1-IR microglia. The data are presented as mean ± SEM of six individual experiments. *** *p* < 0.001, CTL vs. MPTP. * *p* < 0.05, MPTP vs. BCP + MPTP. *** *p* < 0.001, CTL vs. AM630 + BCP + MPTP treatment.

Regarding the effect of BCP on astrocyte and microglia activation in the striatum, markedly activated astrocytes (Figure 8) and microglia (Figure 9) were observed in the mice that were administered MPTP (30 mg/kg, i.p.), as evidenced by a significant increase in GFAP-IR cell numbers (257% compared with that of the control mice) and IBA-1-IR cell numbers (97.4% compared with that of the control mice). Treatment with 10 mg/kg BCP suppressed the MPTP-induced increase in GFAP-IR cell numbers and IBA-1-IR cell numbers by 323.5% and 32%, respectively, (*p* < 0.01) compared with the effects of MPTP treatment alone. The protective effect of BCP against astrocyte and microglia activation was fully reversed by pretreatment with 3 mg/kg AM630. Systemic administration of BCP alone did not significantly affect the number of GFAP-IR or IBA-1-IR cells (Figure 8 and Figure 9).

### 2.4. β-Caryophyllene Inhibits the Levels of Inflammatory Cytokines in the Nigrostriatal System

**Figure 10:**
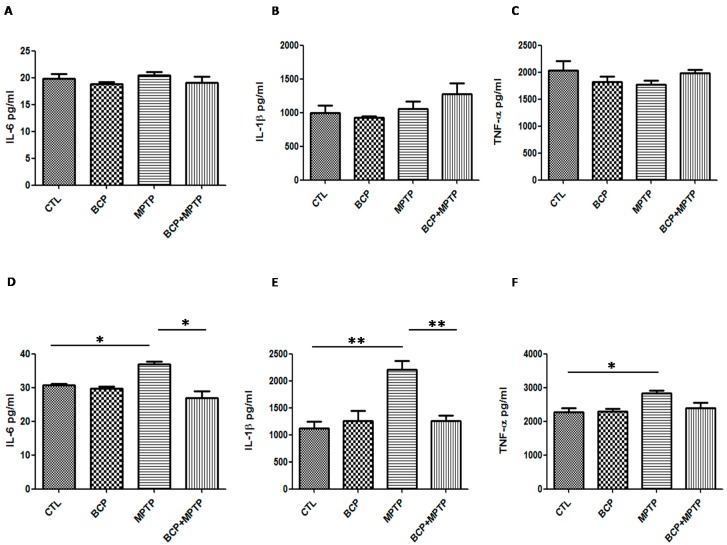
Protein levels of inflammatory cytokines in the nigrostriatal system. Graphsshowing the mean ± SEM of three individual experiments of the protein levels of IL-6, IL-1β, andTNF-α in the striatum (**A**–**C**) and the substantia nigra (**D**–**F**) determined using the indirect ELISA method. **IL-6**: * *p* < 0.05, CTL vs. MPTP treatment. * *p* < 0.05, MPTP treatment vs. BCP + MPTP treatment. **IL-1β**: ** *p* < 0.01, CTL vs. MPTP treatment. ** *p* < 0.01, MPTP treatment vs. BCP + MPTP treatment. **TNF-α**: * *p* < 0.05, CTL vs. MPTP treatment.

Basal protein levels of IL-6 (30.6 ± 1.1 pg/mL in the SN and 19.8 ± 1.8 pg/mL in the STR), IL-1β (1119.0 ± 255.8 pg/mL in the SN and 995.8 ± 194.1 pg/mL in the STR) and TNF-α (2270.0 ± 223.6 pg/mL in the SN and 2039.0 ± 346.8 pg/mL in the STR) were measured by the Luminex MagPix cytokine assay specific for mice (Figure 10). In mice treated with MPTP (30 mg/kg, i.p.), IL-6 (36.9 ± 1.7 pg/mL in the SN), IL-1β (2207 ± 326.1 pg/mL in the SN) and TNF-α (2826 ± 149.5 pg/mL in the SN) protein levels in the substantia nigra increased significantly when compared with those of the control mice (Figure 10). 

In mice treated with BCP(10 mg/kg, i.p.) and MPTP, IL-6 (26.9 ± 3.9 pg/mL) and IL-1β (1261 ± 192.5 pg/mL) levels decreased but TNF-α levels (2401 ± 257 pg/mL) remained the same compared to those of the control mice (Figure 10). Furthermore, the cytokine levels were unchanged in the striatum. Systemic administration of BCP alone did not significantly affect the levels of inflammatory cytokines.

## 3. Discussion

Various experimental evidence has shown that the endocannabinoid system is a target of pharmacological regulation for the treatment of PD [25]. In the pathology of PD, neuronal death and injury in the SNpc is caused by an inflammatory process largely mediated by the activation of glial cells. A very constant pathogenic condition in PD is oxidative damage leading to the degeneration of dopaminergic cells through the generation of reactive oxygen species (ROS), which ultimately potentiates the MPTP-induced neurotoxicity. It is well known that inflammatory mediators, such as nitric oxide (NO), chemokines and cytokines, play an important role in neuronal cell damage associated with glial cells, which is a crucial neuroinflammatory process involved in the initiation and development of neurodegenerative disorders such as PD [26]. Recently, it has been shown that the use of phytocannabinoid drugs can regulate the activation of glial cells and exert a neuroprotective effect against neuronal damage induced by MPTP, which constitutes an attractive framework for the development of neuroprotective agents against PD. A study published by Gertsch et al., showed that BCP selectively binds the CB2R [27]. Specifically, CB2R ligands, which have been shown to inhibit carrageen-induced mouse paw oedema [28], act on primary afferent neurons to inhibit nociception [29] and play a protective role in hepatic ischaemia reperfusion injury [30]. The use of BCP could offer this neuroprotective function without the psychotropic effects that occur when the CB1R is activated. This suggests that this selective CB2R agonist could potentially be a neuroprotective agent for neurodegenerative diseases, such as PD. Moreover, the endocannabinoid system modulates inflammatory responses through microglia and astroglia function, either by a receptor-dependent or an independent mechanism [31,32]. There are several reports that describe increased CB2R expression in the brain under pathological conditions, mainly in glial cells, which represents a potential therapeutic target for the inhibition of MPTP-induced neuronal damage [16,33,34]. In agreement with previous studies, MPTP administration in rodents produces a robust glia reaction, characterized by activated microglia and reactive astrocytes in the ventral midbrain. A similar reaction has been reported in the brain of PD patients [35]. As expected, in our experimental model, we observed enhanced immunoreactivity of GFAP-labelled astrocytes and IBA-1-labelled microglia in the SN and striatum of MPTP-treated mice, suggesting that reactive glial cells may play a key role in brain regions damaged by MPTP. In addition, we found that systemic administration of BCP significantly alleviated the MPTP-induced perturbed behavioural symptoms, improved the compromised motor coordination, protected against the degeneration of dopaminergic neurons, and suppressed the production of proinflammatory cytokines, including IL-6 and IL-1β, in the SN. In this sense, several lines of evidence suggest microglia-derived proinflammatory cytokines may be involved in nigrostriatal DA neuronal cell death. Studies of post-mortem PD subjects and models of induced PD show the presence of activated glial cells expressing proinflammatory cytokines, such as IL-1β and TNF-α, in the SN. IL1-β and TNF-α released from glial cells lead to intracellular death-related signalling pathways in the MPTP model of PD. This is comparable tofinding described that activated microglia-derived IL-1β, IL-6 and TNF-α participated in DA neuronal cell death in an MPTP murine model of PD [36]. Additionally, our ELISA data showed that the activation of CB2R through the administration of BCP attenuated the MPTP-induced increases in IL-1β and IL-6 in the SN. Price and colleagues showed that WIN55,212-2 prevents dopaminergic cell death induced by MPTP through the inhibition of microglial cells [15]. These results were reversed by treatment with the CB2R antagonist JTE in MPTP-treated mice, indicating the possible involvement of the microglia CB2R. This is in accordance with our observations that the CB2R antagonist AM630 inhibited the neuroprotective effect of the selective CB2R agonist BCP in MPTP-treated mice. These results demonstrate that it is possible that the activation of the CB2R, at least in part, participates in DA neuronal cell survival through the inhibition of glia activation in an MPTP murine model, although further studies are required to determine the underlying mechanisms. Moreover, the work done by Chung in 2010 demonstrated that the cytokine IL-1β directly participates in MPTP-induced cell death. In this work, the authors also demonstrated that the activation of the CB1R reduces the expression levels of IL-1β in the SN of MPTP-treated mice and that this neuroprotective action was reversed after the administration of specific antagonists to the CB1R and CB2R [6]. These results demonstrate that the activation of cannabinoid receptors can induce a phenotype change of M1 (IFN-γ, TNF-α and IL-2) to M2 (IL-4 and IL-5) through a CB2 receptor-dependent mechanism mediated by anti-inflammatory actions, which can contribute to the neuroprotective effect [34]. An important cytokine in the establishment of the M2 phenotype is IL-10 [37,38]. Anandamide treatment induced an increase in the production of IL-10, mediated by the CB2R, as determined by the fact that blocking this receptor with a specific antagonist (SR2SR145228) reversed this effect. Additionally, use of the CB2R specific agonist JWH-133 produced similar effects as the AEA treatment.

During an inflammatory process astrocytes and microglia cells, in addition to secreting mediators of inflammation, such as IL-6, IL-1β, TNF-α, nitric oxide and reactive oxygen species that exert neurotoxic effects during pathological states [39], which can contribute significantly to the neurodegenerative process underlying PD [40,41]. In addition, these cells are able to release endocannabinoids to combat various neuropathological conditions [42] and traumatic CNS lesions [43].These neuroinflammatory processes require a delicate balance between the production of pro- and anti-inflammatory cytokines [44] so that inflammation is maintained within appropriate limits.

Many studies have reported that the activity of neurotrophic factors could prevent neurodegenerative disease [45,46]. In particular, glial cell line-derived neurotrophic factor (GDNF) is associated with astrocyte activation in PD [47,48]. These findings suggest that astroglia activation might be beneficial for preventing PD pathologies through the production of neurotrophic factors, such as GDNF and ciliaryneurotrophic factor (CNTF). Recent research also has suggested that the expression of transient receptor potential vanilloid 1 in astrocytes is involved in neuroprotection in PD through the endogenous production of CNTF [49].

Although MPTP causes pathophysiological alterations in mice similar to those reported in idiopathic PD, this neurotoxin produces transient hypokinetic behaviour and inconsistent motor deficits that disappear within a few d of its administration [50,51,52,53,54,55]. One of the important causes of motor dysfunction induced by MPTP is decreased levels of DA in the SN and STR, which is mainly due to a lack of DA synthesis and storage in dopaminergic nerve endings [56]. The connection between the SNpc and the STR is critical for producing controlled and coordinated movement. A decrease in the levels of dopamine in this circuit results in abnormal nerve-firing patterns within the brain that cause uncontrolled and abnormal movements. Von Bohlen (2005), reported that the decrease in dopamine synthesis and storage in the dopaminergic nerve endings causes motor dysfunction in PD, while MPTP-induced TH reduction in the SNpc and STR is correlated with motor dysfunction [57,58]. To reveal behavioural correlates to MPTP-induced lesions, we used the gait, beam and pole performance tests [59]. Our results showed that BCP improved the abnormal behaviours in MPTP-treated mice, as manifested by the observation of a reduced total time required to climb down the pole, prolonged latent periods in the rotarod test, and increased vertical movements. The involvement of the CB2 receptor in the regulation of emotional behaviour suggests that this receptor could be an important therapeutic target for the treatment of anxiety and depressive disorders. In this sense, our results from the pole test showed that animals can generate pessimistic or dangerous thoughts. This was determined because mice treated with BCP showed considerably decreased of the fear caused by the pole height; the animals were very confident at the time of the test, which resulted in decreased time required to perform the test [60]. It is known that the modulation of CB2 receptor activation leads to increased levels of endocannabinoids, such as 2-AG and AEA, which can induce optimistic behaviour in mice. These effects of BCP were observed at a 10 mg/kg dose administered both orally and intraperitoneally. Based on the present results, we suggest that BCP may serve as a therapeutic agent for the alleviation of motor dysfunction by preventing dopaminergic neuronal damage.

However, one of the limitations of this study was that the dopamine levels were not assessed in the striatum and substantia nigra. In the animal studies, the endocannabinoid system has been reported to activate the dopaminergic system and to increase the availability of DA in the striatum. It is known that the DA neurons are key neurotransmitters that play a role in regulating growth, maintenance, and synaptic plasticity.

## 4. Materials and Methods

### 4.1. Animals

All experiments were carried out using male C57BL/6J mice (25–30 g of body weight) that were maintained under a 12:00 h light-dark cycle with food and water available ad libitum. The animals were obtained from Harlan Laboratories (Mexico City). All animal handling and experimentation strictly followed the Guidelines for Care and Use of Laboratory Animals published by the National Institutes of Health and the Guidelines of the Mexican Law of Animal Protection. Animal care and experimental procedures were in accordance with the Mexican Official Norms NOM-062-ZOO-1999 and NOM-033-ZOO-1995. All experimental procedures were approved by the research and ethics committees of the University of Guadalajara in Mexico. We minimized the number of mice used and their suffering or pain as much as possible.

### 4.2. Drug Administration Schemes

Animals were randomly divided into four groups (*n* = 6/group). The first group of mice was saline-treated and served as a control group. The second group of mice was treated with MPTP following the subchronic scheme, which consisted of MPTP hydrochloride (30 mg/kg, i.p.; Sigma-Aldrich, St. Louis, MO, USA) being administered daily for five consecutive days. The MPTP administration conditions and dosage were selected based on previous studies [61] in which dopamine depletion, apoptosis, inflammatory response, and oxidative damage were demonstrated in mice subjected to MPTP under the same experimental conditions. The third group of mice was treated with BCP (10 mg/kg, i.p.) for five d. The fourth group of mice was pretreated with BCP for five d, and one day after the last administration of BCP, the animals were treated with MPTP (30 mg/kg, i.p.) for 5 consecutive days. The fifth group of mice received an administration of the CB2R antagonist AM630 (3 mg/kg, i.p., SML0327 Sigma-Aldrich) 30 min prior to an injection of another CB2R agonist, BCP. The control mice were injected with either a CB2 agonist and an antagonist alone or a vehicle. The animals were sacrificed 3 days after treatment was completed. To evaluate the effects of BCP administered orally, a group of animals was administered BCP (10 mg/kg) through an oral gavage for 5 consecutive days, and one day after the last administration of BCP, the animals were treated with MPTP (30 mg/kg, i.p.) for 5 consecutive days.

### 4.3. Behavioural Studies

Behavioural tests were conducted three days after the last administration of each treatment.

#### 4.3.1. Pole Test

Bradykinesia is measured by determining the amount of time it takes an animal to turn around and fully descend a pole according to a method described by Matsuura et al. (1997). Mice were first acclimated to the pole (1 cm diameter, 50 cm height) in 10 trials, with each trial being separated by 120 s. In the first trial, the animals were limited to 300 s, and subsequent trials were limited to a 120 s maximum time limit. The three best scores were averaged for each mouse [62,63].

#### 4.3.2. Gait Test

The gait test was performed according to the method established by Fernagut et al. The apparatus was 7 cm wide, 12 cm high and 60 cm long, finished in a dark box, and placed on an interchangeable part [64]. The forepaw of the subject mouse was wet with non-toxic blue ink, and the mouse was placed at the opposite end of the runway, which was covered with a strip of paper (7 cm wide and 60 cm long). The stride length of the forelimb was measured manually as the distance between two forepaw prints. The three longest strides were measured. The first, second, and last paw prints were excluded because the velocity changed during the run.

#### 4.3.3. Beam Test

This test uses a device that the mice escalate consisting of a 1-metre long acrylic beam placed at a 15-degree angle. The test mice were placed at the upper end of the beam, which was the box-home stimulus. Initially, the mice were trained to climb a beam that was 12-mm wide for two d prior to beginning the management schemes; the test was initiated by changing the width of the beam to 6-mm wide. Mice were placed at the bottom of the beam and allowed to walk to reach the upper end while recording the time it took to arrive, with a maximum limit of 120 s. After 120 s, if the mouse had not reached its box-home manually, it was removed and placed in its box and received a rating of 120 s. The results are expressed as the average total time (s) it took for the mice from each experimental treatment group to run the test.

### 4.4. Measurement of the IL-1β, TNF-α and IL-6 Cytokines

Analyte levels in cerebral homogenateswere measured using a commercially available Luminex MagPix cytokine (Bio-Rad, Hercules, CA, USA) assay specific for mice according to the manufacturer’s instructions. The MagPix assay uses a red LED instead of a laser to excite the dyes in the beads (6.5-µm magnetic beads) for the identification of specific analytes and a green LED to excite the reporter ligand. Acquisition of the fluorescent signal was achieved by a CCD imager. The concentration of the analytes was determined using Bio-Plex® 200 System and Bio-Plex™ Human Cytokine Standard 27-Plex, Group I (Bio-Rad). The assays were run in triplicateof three individual experimentsand the concentrations are expressed in pg/mL based on a standard curve.

### 4.5. Immunohistochemistry of Tyrosine Hydroxylase, GFAP and Iba

Three days after the last treatment was administered, the mice (*n* = 5 per group) were anaesthetized (100 mg/kg ketamine and 15 mg/kg xylazine, i.p.) and an intracardiac perfusion was performed with 0.1 M PBS solution followed by 4% paraformaldehyde. After the perfusion, the brains were removed, kept in fixative solution for 24 h, and subsequently washed 3 times with 0.1 M PBS. Coronal 35-µm sections were sliced on a vibratome (Leica VT1000E; Leica Microsystems, Wetzlar, Germany) to obtain the substantia nigra pars compacta (SNpc) and striatum (STR) regions. For morphological analysis of the STR and SNpc, coronal slices were cut at the bregma and −3.3 mm from the bregma, respectively, according to the protocol provided by Paxinos and Franklin [65]. Six tissues were collected from each individual brain, with 175 µm between each slice. According to the coordinates mentioned above, slices were selected at the same level in all animals to obtain a uniform analysis using the basic principle of fractionation from the caudal face area. Analysis of the results was performed to obtain the average number of marker-positive cells per field (445-µm) at 40× magnification cells.

### 4.6. Tyrosine Hydroxylase Assessment

Samples were rinsed in 0.1 M PBS and then incubated in pre-warmed sodium citrate buffer (pH = 6) for 10 min. Next, the samples were washed three times with 0.1 M PBS and then incubated with 1% hydrogen peroxide to block endogenous peroxidase staining. The slices were then washed first with 0.3% Triton X-100 in 0.1 PBS, followed by an additional two washes with 0.1% Triton X-100 in 0.1 PBS. The samples were then incubated with 10% goat serum and 0.1% Triton X-100 in 0.1 PBS for 45 min, followed by incubation for 72 h at 4 °C in the same buffer solution containing a tyrosine hydroxylase antibody (Ab152 Merck Millipore, Billerica, MA, USA). The samples were then washed (4 × 5 min) in 0.1 M PBS and incubated for 2 h at room temperature in the dark with an anti-rabbit IgG (BA1000, Vector Labs, Peterborough, UK) antibody at a dilution 1:500. An Elite ABC kit (Vector, PK6000) system was applied in the dark at a dilution of 1:200. The samples were detected with 3′3 diaminobenzidine (DAB kit, Vector Labs, Peterborough, UK). To determine the TH-immunohistochemistry in the striatum, the sections that were −3.3 mm relative to the bregma were selected to quantify the optical densities of the TH-immunoreactive fibres according to a previously described method [56]. TH-immunoreactive fibre density was measured in 100 × 100 μm square images of the striatum using an image analyser (Multiscan, Fullerton, CA, USA).

### 4.7. GFAP and Iba Assessment

Other tissue sections were processed to identify astrocyte and microglial cells. To identify astrocytes immunocytochemically, washes and incubations were performed on free-floating tissue sections while being moderately shaken. Briefly, tissue sections were incubated in 1% hydrogen peroxide to block endogenous peroxidase staining. Next, the slices were washed (4 × 5 min) with 0.1 Triton X-100 in 0.1 PBS and then incubated for 45 min with 0.1 Triton X-100 in 0.1 PBS and 10% goat serum. The slices were incubated in this same buffer overnight at 4 °C with an anti-glial fibrillary acidic protein (GFAP) polyclonal antibody (DAKO, Glostrup, Denmark) at a dilution of 1:400 and an anti-rabbit IgG secondary antibody (BA1000, Vector Labs). Then, complex incubation using the Elite ABC kit (Vector, PK6000) was performed in the dark for 2 h at a dilution 1:200. After being washed with 0.1 M PBS, the tissue sections were detected with 3′3-diaminobenzidine.

Histochemical staining of microglia was performed as follows: To inhibit peroxidase activity, sections were incubated for 20 min with 1% H_2_O_2_. Tissues were rinsed in 0.1 M PBS (4 × 5 min) and incubated for 45 min in 0.1 M PBS and 10% goat serum. They were then incubated overnight at 4°C with lectin from Bandeiraea BS-1.

## 5. Statistical Analysis

Statistical analysis was performed using GraphPad Prism5 software (GraphPad Software, Inc., La Jolla, CA, USA). Data are expressed as the means ± SD. The multiple comparison one-way analysis of variance and Tukey’s HSD *post hoc* tests were used for statistical analysis. *p*-values ≤ 0.05 were considered statistically significant.

## Abbreviations

PD	Parkinson’s disease
BCP	β-caryophyllene
CB1R	type 1 cannabinoid receptor
CB2R	type 2 cannabinoid receptor
MPTP	1-methyl-4-phenyl-1,2,3,6-tetrahydropyridine
GFAP	glial fibrillary acidic protein
TH	tyrosine hydroxylase
CNS	central nervous system
NO	nitric oxide
ROS	oxygen species
GDNF	line-derived neurotrophic factor (GDNF)
CNTF	ciliary neurotrophic factor
SNpc	substantia nigra pars compacta
STR	striatum
