# Correction: Viveros-Paredes et al. Neuroprotective Effects of β-Caryophyllene against Dopaminergic Neuron Injury in a Murine Model of Parkinson’s Disease Induced by MPTP. *Pharmaceuticals* 2017, *10*, 60

**DOI:** 10.3390/ph17101374

**Published:** 2024-10-16

**Authors:** Juan M. Viveros-Paredes, Rocio E. González-Castañeda, Juerg Gertsch, Veronica Chaparro-Huerta, Rocio I. López-Roa, Eduardo Vázquez-Valls, Carlos Beas-Zarate, Antoni Camins-Espuny, Mario E. Flores-Soto

**Affiliations:** 1 Departamento de Farmacobiología CUCEI, Universidad de Guadalajara, Guadalajara 44430, Mexico; jviveros99@hotmail.com (J.M.V.-P.); rlopezroa@gmail.com (R.I.L.-R.); 2 Laboratorio de Microscopía de Alta Resolución, Departamento de Neurociencias, Centro Universitario de Ciencias de la Salud, Universidad de Guadalajara, Guadalajara 44340, Mexico; roglezca@yahoo.com.mx; 3 Institute of Biochemistry and Molecular Medicine, NCCR Trans Cure, University of Bern, CH-3012 Bern, Switzerland; gertsch@ibmm.unibe.ch; 4 Laboratorio de Neurobiología Celular y Molecular, Centro de Investigación Biomédica de Occidente (CIBO), Instituto Mexicano del Seguro Social, Guadalajara 44421, Mexico; veronicach73@mail.com; 5 Laboratorio de Inmunodeficiencias y Retrovirus Humanos, Centro de Investigación Biomédica de Occidente, Instituto Mexicano del Seguro Social, Guadalajara 44421, Mexico; evazquez@cencar.udg.mx; 6 Laboratorio de Regeneración y Desarrollo Neural, Instituto de Neurobiología, Departamento de Biología Celular y Molecular, CUCBA, Universidad de Guadalajara, Guadalajara 44340, Mexico; carlosbeas55@gmail.com; 7 Unitat de Farmacologia i Farmacognòsia, Facultat de Farmàcia i Ciencias de l’Alimentació, Universitat de Barcelona, 08028 Barcelona, Spain; camins@ub.edu; 8 Biomedical Research Networking Center in Neurodegenerative Diseases (CIBERNED), 28031 Madrid, Spain

In the original publication [1], there was a mistake in Figure 5 as published. We used the wrong image for “BCP” in Figure 5A. The corrected Figure 5 appears below. The authors state that the scientific conclusions are unaffected. This correction was approved by the Academic Editor. The original publication has also been updated.

## Figures and Tables

**Figure 5:**
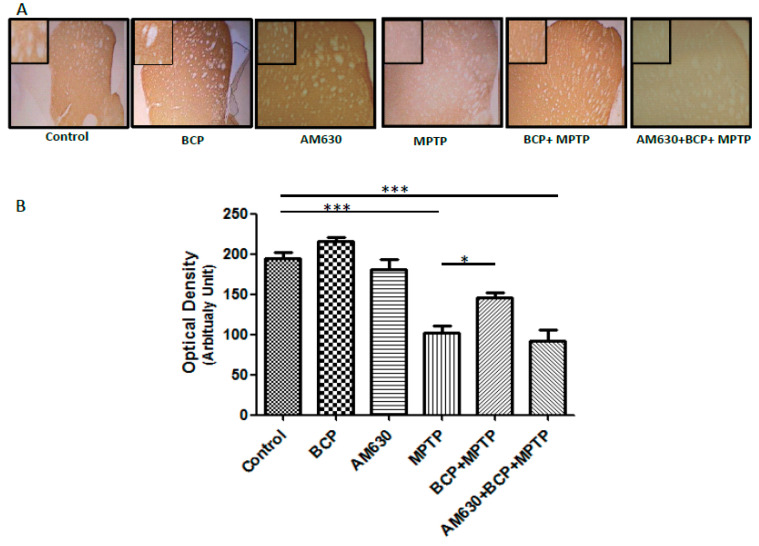
BCP treatment attenuates MPTP-induced nigrostriatal dopaminergic neuronal damage. C57BL/6 mice were treated with MPTP for 5 days (30 mg/kg, i.p.). The mice were sacrificed on the 3rd day after MPTP injection after being subjected to behavioural tests. Photomicrographs of representative STR (**A**) sections stained with an antibody against TH. Reduced activity of TH-neurons was observed in the MPTP-treated mice, which was partially prevented by treatment with BCP. The number of TH-positive fibres in the STR (**B**) was expressed as the mean ± SEM of six individual experiments. *** *p* < 0.001, CTL vs. MPTP treatment. * *p* < 0.05, MPTP treatment vs. BCP + MPTP treatment. *** *p* < 0.001, CTL vs. AM630 + BCP + MPTP treatment.
